# The microneme protein1 (MIC1) of Chinese 1 *Toxoplasma* regulates pyroptosis through the TLR4/NLRP3 pathway in macrophages

**DOI:** 10.1186/s13071-024-06584-z

**Published:** 2024-11-29

**Authors:** Wenze Sun, Fan Zhang, Jinjin Zhu, Yanxia Yu, Yang Wang, Qingli Luo, Li Yu

**Affiliations:** Department of Microbiology and Parasitology; Anhui Province Key Laboratory of Zoonoses, School of Basic Medical Sciences, Anhui Medical University, Hefei, 230032 People’s Republic of China

**Keywords:** TgMIC1, *Toxoplasma gondii*, Pyrptosis, TLR4, NLRP3

## Abstract

**Background:**

TgMIC1, a soluble adhesion protein that typically facilitates parasite invasion, exhibited varying expression levels among distinct virulence strains of Chinese 1 *Toxoplasma*. This study aims to explore its role in immunological regulation and its association with diverse postinfection outcomes in *Toxoplasma* infection.

**Methods:**

First, the mic1 knockout strain Wh3Δ*mic1* was generated and assessed for its virulence and proliferative capacity. Subsequently, the serum inflammation levels were examined in mice infected with Wh3Δ*mic1*, Wh3, and Wh6. Furthermore, rMIC1 and rMIC1-T126A/T220A, which lack binding sites to *N*-glycan in TLR4, were produced for coculture with bone marrow-derived macrophages (BMDMs) to investigate their impact on pyroptosis.

**Results:**

Our data showed Wh3Δ*mic1* exhibited a significant reduction in invasion efficiency, limited growth, and attenuated inflammatory responses in mice. Additionally, it displayed a decreased capacity to induce pyroptosis when compared with Wh3-infected BMDMs. Moreover, rMIC1 but not rMIC1-T126A/T220A was found to be able to upregulate NOD-like receptor pyrin domain-containing protein 3 (NLRP3) and activate GSDMD and caspase-1 in BMDMs but not in TLR4^−/−^ and NLRP3^−/−^ BMDMs.

**Conclusions:**

TgMIC1 is implicated in both parasite invasion and the modulation of macrophage pyroptosis via the TLR4/NLRP3 pathway. This investigation indicates that TgMIC1 serves diverse functions in *Toxoplasma gondii* infection, thereby enhancing comprehension of the immune regulatory mechanisms of the parasite.

**Graphical Abstract:**

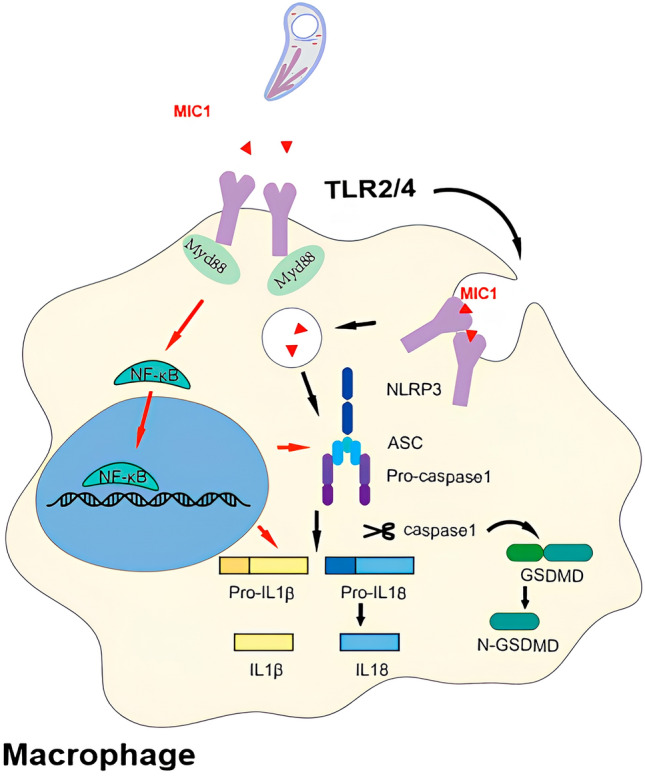

**Supplementary Information:**

The online version contains supplementary material available at 10.1186/s13071-024-06584-z.

## Background

*Toxoplasma gondii* is an obligate intracellular parasite capable of invading the nuclei of virtually all nucleated cells in warm-blooded animals. While infection with *T. gondii* is typically benign for healthy adults in most cases, it can pose significant risks to fetuses and individuals with weakened immune systems. The severity of the consequences also depends on the genetic type of *T. gondii* SPS: refid::bib1|bib2 [1, 2]. T. gond*ii* exhibits an unusual population structure due to its global distribution and complex life cycle. Based on polymerase chain reaction followed by restriction fragment length polymorphism (PCR–RFLP) analysis, the population structure of *T. gondii* has been classified into three types: type I, type II, and type III, which are referred to as the archetypal clonal lineages [3]. These three clonal lineages are predominant in Europe and North America. However, in China, the dominant genotype is entirely distinct from the archetypal clonal lineages and is referred to as Chinese 1 (ToxoDB#9) [4].

There is a noticeable difference in virulence between *T. gondii* strains with distinct genotypes, which has been influenced by differentially expressed effector proteins. Previous experiments have consistently shown that type I strains are highly virulent, while type II and type III strains have lower virulence levels [5]. Notably, while both the highly virulent TgCtwh3 (Wh3) strain and the low virulent TgCtwh6 (Wh6) strain belong to the Chinese 1 genotype, they display different levels of virulence in mice [6]. Extensive research has been conducted to investigate the differences in virulence between these archetypal clonal lineages, and various virulence factors have been identified, including ROP5/18, ROP16, and GRA15 [7–14]. In the initial investigation of the differing virulence of Wh3 and Wh6, RNA sequencing (RNA-seq) was utilized. The analysis showed no significant differences in the aforementioned virulence factors between the two strains. However, a notable disparity in the expression of microneme protein 1 (MIC1) was observed [15]. These findings suggest that TgMIC1 may play a role in modulating the virulence of the Chinese 1 strain.

TgMIC1, a 49-kDa soluble protein, serves as the central component of the complex by interacting concurrently with TgMIC4, TgMIC6, and host cells [16, 17]. Moreover, TgMIC1 is necessary for the successful transit of TgMIC4 and TgMIC6 from the endoplasmic reticulum (ER) and the Golgi apparatus, the initial compartments of the secretory pathway [18–21]. The TgMIC4/TgMIC1/TgMIC6 complex is the first to be identified and one of the most extensively investigated microneme complexes in the parasite. It has been shown to be critical in invasion, along with TgAMA1- RON2/RON4/RON5/RON8, TgMIC3/TgMIC8, and TgMIC2/M2AP [22–25]. Upon secretion outside the parasite, TgMIC6 anchors the complex to the surface and interacts with the actin–myosin system to facilitate its gradual transfer toward the rear, enabling penetration of the host. After the invasion is completed, rhomboid (ROM) 4, ROM5, and microneme processing protease 1 (MPP1) hydrolyze the intramembrane region of the C-terminal transmembrane domain, leading to cleavage of the complex from the surface of the parasite and disconnection from the host cell [26]. Although earlier studies have clearly highlighted the roles of TgMIC1 and its complex TgMIC4/TgMIC1/TgMIC6 in invasion, the functionality of the three independent components of the complex, including TgMIC1, after their release remains poorly understood.

In the present study, we found Wh3Δ*mic1* not only exhibited a significant reduction in invasion efficiency but also decreased capacity to induce pyroptosis. Moreover, rMIC1 induced pyroptosis in bone marrow-derived macrophages (BMDMs) through the TLR4/NLRP3 pathway. The data suggest that the TgMIC1 is involved both in the invasion of *T. gondii* and in immune regulation.

## Methods

### Parasites and cells

Tachyzoites of the *T. gondii* Wh3 and Wh6 were maintained in human foreskin fibroblast (HFF) cells using Dulbecco’s modified Eagle medium (DMEM, Gibco, USA) supplemented with 2% fetal bovine serum (FBS, BI, Israel). HFF and Vero cells were routinely maintained in DMEM supplemented with 10% FBS, 100 μg/ml penicillin and 100 μg/ml streptomycin (Sigma, USA) and cultured in a 37℃ and 5% CO_2_ incubator. The HFF cell line was obtained from long-term preservation in our laboratory.

### Construction of Wh3 *mic1* gene knockout strains

The *mic1* gene knockout strains were constructed as described in previous study. UPRT-targeting guide RNA (gRNA) in pSAG1: CAS9-U6::sgUPRT (Addgene plasmid no. 54,467) was replaced with MIC1 targeting gRNA using Q5 mutagenesis kit (New England Biolabs, Ipswich, MA). Homologous templates of MIC1 were amplified from the genomic DNA of TgCtwh3 and, along with the DHFR*-Ts, were cloned into pUC19 using ClonExpress Multis One Step Cloning Ki (Vazyme biotech, Nanjing, China). The pSAG1::CAS9-U6::sgMIC1 and donor DNA fragments were cotransfected into freshly harvested tachyzoites of Wh3 strain by electroporation. Parasites were selected with pyrimethamine for DHFR-TS. Diagnostic polymerase chain reactions (PCR)s (RCR1, PCR2, and PCR3) were performed with genomic DNA as a template to confirm the *mic1* gene was knocked out. The primers used in the study are presented in Table 1.

**Table 1:** Primers used in the construction of Wh3Δ*mic1*

Primers	Sequence	Used for
5′-MIC1-guide RNA (gRNA)	TGGTAGAAGGCAATAAATAACTGCAAGCTTGGCGTAATCATGGTC	Q5 mutagenesis changing the gRNA in pSAG1::CAS9-U6::sgUPRT to gRNA-MIC1
3′-MIC1-gRNA	ATCTCCTGCTCTGTATTGCTTGAATTCACTGGCCGTCGTTTT	
UpMIC1 F	AAAACGACGGCCAGTGAATTCTATCGGAATGTAGTGGTGCTCACCA	To produce UpMIC1 PCR product for making pMIC1:DHFR-I
UpMIC1 R	GGGGGGTGAAAATCGAATGACATGCAAATCCGTTCTAGGTCAACTACA	
DHFR-TS F	TGTAGTTGACCTAGAACGGATTTGCATGTCATTCGATTTTCACCCCCC	To produce UpMIC1 PCR product for making pMIC1:DHFR-I
DHFR-TS R	CCACAAGCATGCTCCAAAGAGTACTATAAAGGAATTCATCCTGCAAGTGCAT	
DnMIC1 F	ATGCACTTGCAGGATGAATTCCTTTATAGTACTCTTTGGAGCATGCTTGTGG	To produce UpMIC1 PCR product for making pMIC1:DHFR-I
DnMIC1 R	GACCATGATTACGCCAAGCTTGCAGTTATTTATTGCCTTCTACCAGAAGTC	
5′-UpMIC1	TCGACAACGAATGACACACAGGAAC	PCR1
5′-InDHFR	TCGACAACGAATGACACACAGGAAC	
3′-InDHFR	CATGTGGCATTTCACACAGTCTCAC	PCR2
3′-DnMIC1	GATCTACTGGCACTCGCCGA	
5′-InMIC1	AAGGCGGACTTCGTAAAATGTGT	PCR3
3'-InMIC1	TACCCACTCCTTGGAAACTAACCTCT	
5′pUC19	GAATTCACTGGCCGTCGTTTT	To make linearized pUC19 plasmid
3′pUC19	AAGCTTGGCGTAATCATGGTC	

### Virulence assay

Specific pathogen-free (SPF) female C57BL/6 mice (6 weeks of age) were infected with 1000 freshly harvested tachyzoites of Wh3 WT, Wh6 WT, and Wh3Δ*mic1*, respectively. All mice were monitored daily until death and the survival rate was recorded.

### Invasion assay

Vero cells (Cell Bank of Type Culture Collection of Chinese Academy of Science, Shanghai, China) monolayers were grown on coverslip placed in wall of a six-well plate at a density of 10^5^ cells per well. Then, the cells were challenged with fresh tachyzoites of Wh3 WT, Wh6 WT, and Wh3Δ*mic1* after 2 h of culture. Using PBS wash away non-adherent parasites after 1 h. Then fixation with 4% paraformaldehyde (PFA) for 20 min. Adherent external parasite were detected using rabbit anti-*T. gondii* glide-associated protein 45 (TgGAP45) antibodies (1:2000, gifted by Doctor Yonggen Jia, Beijing Friendship Hospital, Capital Medical University). Then, the cells were incubated by secondary anti-rabbit antibodies coupled to Alexa Fluor 594 (1:1000, Invitrogen, USA). All parasites including intracellular and adherent external parasites were labeled with anti-GAP45 antibodies after permeabilization with 0.1% Triton-100 (Sigma, USA) for 30 min. Then cells were incubated by secondary anti-rabbit antibodies coupled to Alexa Fluor 488 (1:1000, Invitrogen, USA). The date was compiled from three independent experiments. For each experiment, at least 100 parasites were counted and each sample randomly select three fields. The invasion efficiency was calculated as the number of successfully invaded parasites (green) divided by the total number of parasites (green and yellow).

### Intracellular growth assay

Vero cells were infected with freshly harvested tachyzoites of Wh3 WT, Wh6 WT, and Wh3Δ*mic1* after 24 h of culture. At 36 h postinfection, the cells were washed three times with PBS. The coverslips were then fixed with 4% PFA and subjected to the Wright–Giemsa staining method before observation under a microscope. Fifty parasitophorous vacuoles (PVs) were randomly selected, and the number of *T. gondii* tachyzoites within each PV was counted. For each condition, 100 vacuoles were counted in three independent replicates. The Vero cell line was obtained from long-term preservation in our laboratory.

### Plaque assay

Vero cells were plated in a 24-well plate. After the cells were completely attached and covered to 100%, the freshly lysed tachyzoites were washed and counted and then added to the wells according to the cell-to-tachyzoite ratio of 3:1. The groups were divided into Wh3 WT, Wh6 WT, and Wh3*Δmic1*, which were incubated at 37 ℃ for 7 days. After incubation, the liquid was removed from the wells and washed with cold PBS three times, each for 5 min. The wells were dried, then 500 μl of 4% paraformaldehyde were added to each well and fixed for 10 min. After fixing, the paraformaldehyde was removed and the wells were washed with 1 ml of cold PBS three times, each for 5 min. The residual liquid was absorbed, then 400 μl of crystal violet dye solution was added to each well and stained at room temperature for 20 min. After staining, it was rinsed with clean water and pictures were taken of the plaques once they were dry. The number and size of the plaques were determined using Photoshop.

### Expression and purification of recombinant TgMIC1 proteins (rMIC1) and rMIC1-T126A/T220A

*Escherichia coli* BL21 carrying the pET30a-MIC1 plasmid was cultured in Luria–Bertani (LB) medium according to previously established methods [15]. The lysate was subjected to centrifugation at 12,000 rpm for 20 min following sonication on ice to obtain the protein supernatant. Subsequently, the supernatants were purified using a Ni column (Ni Sepharose 6 Fast Flow, GE Healthcare). Removal of lipopolysaccharides (LPS) was achieved using triton-114, and the resulting supernatants were analyzed using SDS-PAGE and the BCA Protein Quantitation Kit (E112-01, Vazyme biotech, China). Construction of the pET30a-MIC1-T126A/T220A variant based on pET30a-MIC1 was accomplished using the Q5 mutagenesis kit, following the protocol described in a previous publication [24]. Details of the primers used are provided in Table 2.

**Table 2:** Primers used in the construction of rMIC1-T126A/T220A

Primers	Forward primer (5′–3′)	Reverse primer (5′–3′)
rMIC1-126	TAAACTGGTTGAAGAAGGTGTGCAGCG	CTCAGGATTTCATGACGGGCCGCATGATTAGAG
rMIC1-220	GTCAGGCTATCGGCTCTGT	GAATACCTTCTTCTTCCGCATAATGGCGTTTATC

### Isolation and culture of BMDMs

All mice were euthanized by spinal cord dislocation method after being anesthetized with intraperitoneal injection of sodium pentobarbital (80 mg/kg) and losing consciousness. Sodium pentobarbital has a narrow safety margin, is potent, and can be formulated as a concentrated solution so that relatively small volumes are needed. Intraperitoneal injection is relatively simple and quick to perform, allows the administration of large volumes, and accommodates repeated injections. BMDMs obtained from 6-week-old female C57BL/6, TLR4^−/−^ and NLRP3^−/−^ mice were cultured in DMEM supplemented with 10% FBS and 30% L929 cell supernatant for 7 days as previously described [27]. L929 mouse fibroblast cells were cultured in DMEM with 10% FBS, and the cell supernatant was collected on the fifth day. The positive control group BMDMs were treated with 200 ng/ml LPS (M9524, Abmole, USA) for 5 h, followed by a 10 μM nigericin (M7029, Abmole, USA) challenge for 1 h. BMDMs were infected with fresh tachyzoites of Wh3 WT, Wh6 WT, and Wh3Δ*mic1* (multiplicity of infection, MOI 3) for 12 h. BMDMs were primed with or without *N*-acetylneuraminic acid (NANA, 50 mM/ml) for 1 h, followed by rMIC1 (5 μg/ml) stimulation for 5 h. The culture supernatants were collected for interleukin (IL)-1β quantitation using an enzyme-linked immunosorbent assay (ELISA). GSDMD, caspase1, and NLRP3 in the cell lysis were detected by western blotting (WB).

### RNA extraction and quantitative real-time PCR (qRT-PCR)

Total RNA was extracted from cells and organs using TRIzol (Invitrogen Life Technologies, Carlsbad, CA) and transcribed into cDNA with Evo M-MLV RTase (Accurate Biology, Hunan, China). *T. gondii* loads in mice and mRNA expression levels of pro-IL-1β, caspase-1, NLRP3, and AIM2 in BMDMs were determined using the SYBR Green Pro Taq HS Kit (Accurate Biology, Hunan, China), with normalization to β-actin. The qRT-PCR primers are listed in Table 3.

**Table 3:** Primers used in qRT-PCR

Primers	Forward primer (5′–3′)	Reverse primer (5′–3′)
529	AGGAGAGATATCAGGACTGTAG	GCGTCGTCTCGTCTAGATCG
Pro-IL-1β	GCAACTGTTCCTGAACTCAACT	ATCTTTTGGGGTCCGTCAACT
Caspase-1	ATGCCGTGGAGAGAAACAAG	CCAGGACACATTATCTGGTG
NLRP3	TGCAGAAGACTGACGTCTCC	CGTACAGGCAGTAGAACAGTTC
AIM2	GTCACCAGTTCCTCAGTTGTG	CACCTCCATTGTCCCTGTTTTAT
β-actin	TTCCTTCCTGGGTATGGAAT	GAGGAGCAATGATCTTGATC

### RNA-seq and bioinformatics analysis

Spleen cells from Wh3-infected and noninfected mice were collected, and RNA library sequencing was performed on the Illumina HiseqTM 2500 by Gene Denovo Biotechnology Co., Ltd (Guangzhou, China). The relative expression level was determined with feature counts, and the differentially regulated genes were called by DEseq2 program in the pipeline with fold-change > 2 and padj < 0.05. Bioinformatics analyses including Gene Ontology (GO) and Kyoto Encyclopedia of Genes and Genomes (KEGG) were analyzed using R studio. The datasets generated and analyzed during the current study are available in the NCBI repository, https://www.ncbi.nlm.nih.gov/bioproject/PRJNA1117525.

### Western blot analysis

To detect mature-IL-1β, the culture supernatants of BMDMs were separated on 12% sodium dodecyl sulfate–polyacrylamide gel electrophoresis (SDS–PAGE) gels and transferred to a polyvinylidene difluoride (PVDF) membrane (Bio-Rad). The blots were cut prior to hybridization with antibodies during blotting. Additionally, to detect GSDMD, NLRP3, and caspase-1, the whole cell lysis of BMDMs were separated on 12% SDS–PAGE gels and transferred to PVDF membrane. The primary antibodies used in the experiment include: β-actin recombinant antibody (1: 1000, 81,115–1-RR, Proteintech, China), NLRP3 rabbit mAb (1:1000, 15,101, Cell Signaling Technology, USA), total and cleaved caspase 1 antibody (1:1000, P79884-2R, Abmart, China), total and cleaved IL1b antibody (1:1000, P50520-1R1, Abmart, China), and total and cleaved N-terminal GSDMD antibody (1:1000, P79887R, Abmart, China). Rabbit anti-MIC1 (1:1000) was raised in our lab. Rabbit anti-PRF (1:1000) was gifted by Doctor Yonggen Jia, Capital Medical University. Goat anti-mouse IgG HRP (1:8000, M21001, Abmart, China) and goat anti-rabbit IgG-HRP (1:8000, M21002, Abmart, China) were as secondary antibodies. The grayscale values of each group were analyzed using ImageJ.

### ELISA

The expression of IL-1β in the cell supernatant or the level of IL-1β, IL-18, IL-10, and interferon (IFN)-γ in the serum of mice was detected by ELISA Kits (Proteintech, China). The absorbance was measured at 450 nm. The value of each group was calculated by the standard curve. The concentration of each sample was calculated using ELISA Calc software.

### Hemagglutination inhibition assay

To prepare the hemagglutination inhibition assay, prepare stock solutions of l-fucose (F2252, Sigma, USA), *N*-acetylneuraminic acid (NANA) (A0812, Sigma, USA), d-mannose (M6020, Sigma, USA), *N*-acetylglucosamine (d-GlcNac) (A3286, Sigma, USA), *N*-acetylgalactosamine (d-GalNac) (A2795, Sigma, USA), d-glucose (D-Glc) (G5767, Sigma, USA), and d-galactose (d-Gal) (3455, Sigma, USA), each at a concentration of 100 mM. Perform serial dilutions of each monosaccharide using PBS buffer. For the experimental group, mix 25 μl of rMIC1 protein solution (1 mg/ml) with 25 μl of the different concentrations of monosaccharide solutions at room temperature for 2 h, then add 25 μl of 2% rabbit red blood cells. For the positive control group, mix 25 μl of lectin (1 mg/ml) with 25 μl of PBS, then add 25 μl of 2% rabbit red blood cells. For the negative control group, mix 50 μl of PBS with 25 μl of 2% rabbit red blood cells. Shake on a shaker at 30 rpm for 1 h. After standing at room temperature for 10 min, observe the agglutination of rabbit red blood cells.

### Statistical analysis

All statistical analyses were performed using GraphPad Prism 8.0, and values of *P* < 0.05 were considered statistically significant. All statistical results were performed at least in three independent experiments. The statistical significance of differences was performed using one-way analysis of variance (ANOVA) *t*-test and Dunnett’s multiple comparisons test.

## Results

### Deletion of TgMIC1 decreases the invasive ability of Wh3

**Fig. 1:**
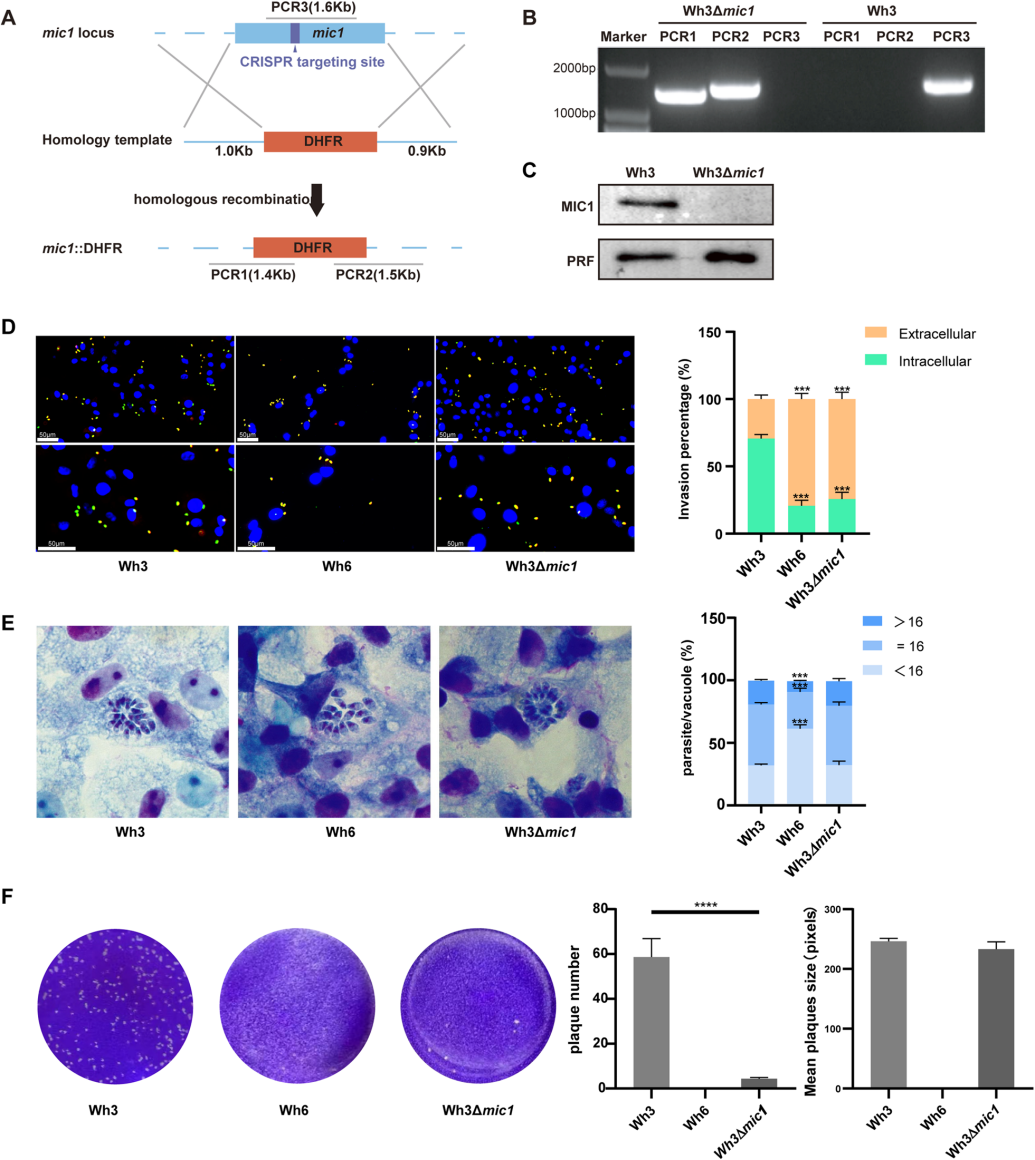
Deletion of TgMIC1 dramatically affects the parasite’s lytic cycle. **A** Schematic diagram of the CRISPR–Cas9 strategy for knocking out the *mic1* gene by inserting the pyrimethamine resistance gene DHFR and primer design for monoclonal PCR identification. **B** PCR identification of the Wh3Δ*mic1* strain of *T. gondii*. As indicated in Fig. 1A, the Wh3Δ*mic1* strain could be amplified using PCR1 and two primers but could not be amplified using the PCR3 primer. **C** Western blot detection of MIC1 expression in Wh3 and Wh3Δ*mic1*. **D** Images of Wh3, Wh6, and Wh3Δ*mic1* tachyzoites invading Vero cells. Red fluorescence was used to label tachyzoites before the transmembrane, and green fluorescence was used to label tachyzoites after the transmembrane. For tachyzoites, the superimposed yellow color of the two colors indicates the tachyzoites outside the membrane, a pure green indicates the tachyzoites in the membrane. One-way ANOVA was used for statistical analyses. Statistical differences are expressed as *P < 0.05 (*n* = 3). **E** The number of parasites per nanovesicle of the Wh3, Wh6, and Wh3Δ*mic1* strains. One-way ANOVA was used for statistical analyses. Statistical differences are expressed as *P < 0.05 (*n* = 3). **F** Number of plaques formed by parasites. The pixels in the mean plaque arca were calculated using Photoshop. Data are presented as the mean ± standard error of the mean (SEM) of each group (*n* = 3) (three independent experiments), and a *t*-test was used for statistical analysis. *P < 0.05

To assess the impact of loss of TgMIC1 function, we used clustered regularly interspaced short palindromic repeats and CRISPR-associated protein 9 (CRISPR–Cas9) to disrupt its gene from the Wh3 tachyzoite. As shown in Fig. 1A, we cotransfected sgMIC1 CRISPR plasmid with a homologous template (DNA fragment generated by PCR amplification) containing pyrimidine-resistant DHFR. The homologous arms on both sides of this homologous template correspond to the 5′UTR and 3′UTR regions directly surrounding the *mic1* CDS sequence. PCR and western blot results showed that the Wh3Δ*mic1* strain was successfully constructed (Fig. 1B, C).

We individually examined the invasion and intracellular replication of the three parasite strains. The invasion efficiency of the Wh3Δ*mic1* strain and Wh6 strain was not significantly different, approximately 20% and 25%, respectively. We did see a significant loss of invasive capability of the knockout, compared with the Wh3 strain, the invasive ability decreased by approximately 70% and 65%, respectively (Fig. 1D). The results of Giemsa staining showed no significant difference in replication ability between the Wh3 and Wh3Δ*mic1* strains, while the replication ability of the Wh6 strain was much lower than that of the Wh3 strains (Fig. 1E). We infected monolayers of Vero cell with Wh3, Wh6, and Wh3Δ*mic1* tachyzoites, allowed the parasites to grow for 7 days, and then quantified the area of plaques formed on the monolayers. Crystal violet staining results showed that the number of plaques formed by Wh3Δ*mic1* were significantly lower than those formed by Wh3 strains (Fig. 1F). These data indicate that MIC1 plays an important role in regulating the invasion and lytic cycle of Wh3 strain.

### Deletion of TgMIC1 attenuates Wh3 virulence in mice

**Fig. 2:**
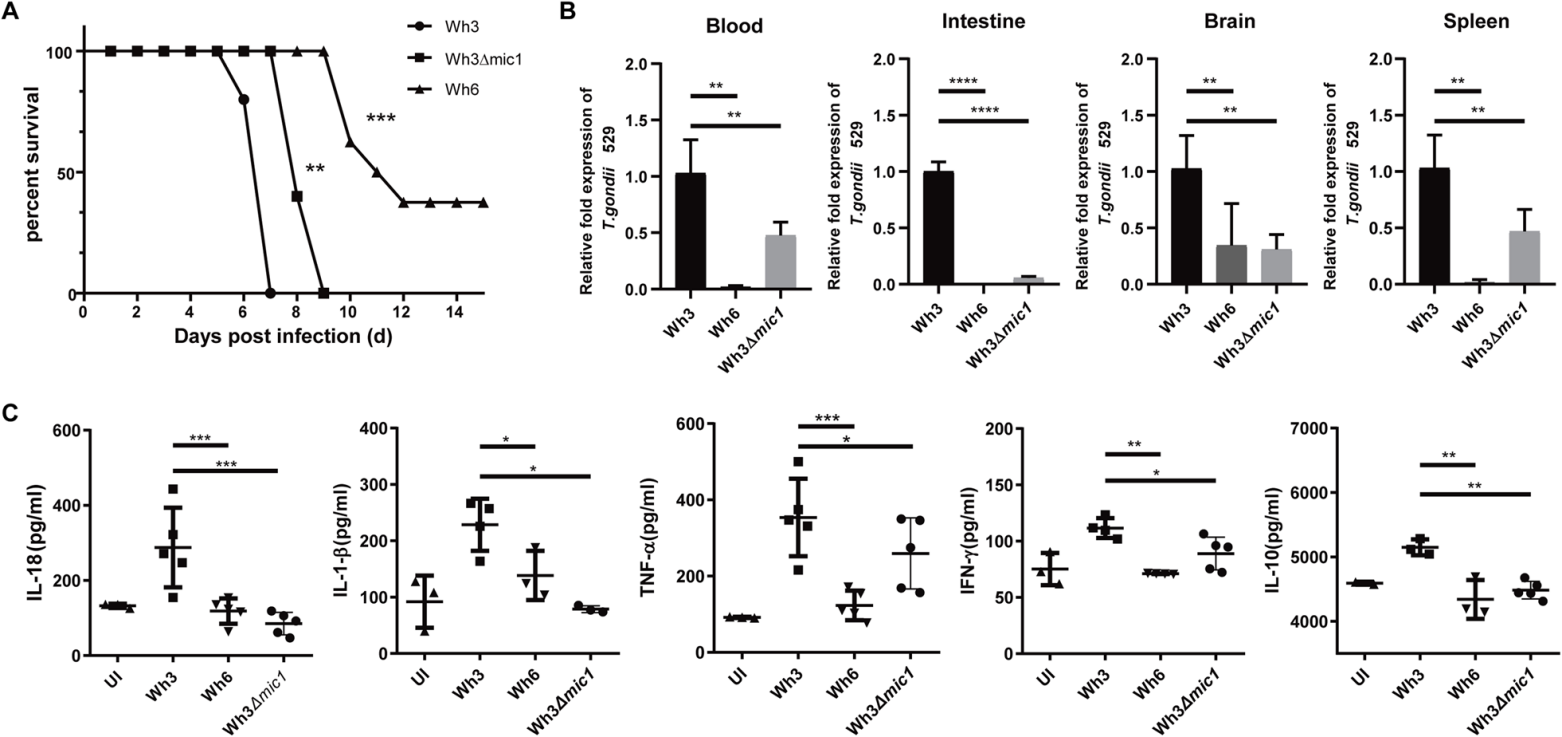
Deletion of TgMIC1 attenuates Wh3 virulence in mice. **A** Evaluation of the virulence of the Wh3, Wh6, and Wh3Δ*mic1* strains using Kaplan–Meier analysis. **B**, **C** C57BL/6 mice were intraperitoneally infected with 1 × 10^3^ freshly harvested tachyzoites of Wh3, Wh6, and Wh3Δmic1 strains (12 C57BL/6 mice were used in each group). On day 5, qRT-PCR was used to detect 529 bp repetitive element (529 bp-RE) to assess the *Toxoplasma* load in the blood, intestine, brain, and spleen, while serum cytokine levels were measured using ELISA. The experiments included at least three mice per group, and statistical significance was set at *P* < 0.05 (*n* = 3). Unpaired *t*-tests were used for statistical analysis. The data are presented as the mean ± standard error of the mean (SEM)

Given the importance of TgMIC1 during Wh3 in vitro infection, we infected C57BL/6 mice by intraperitoneally injection with 1000 Wh3, Wh6, and Wh3Δ*mic1* tachyzoites and analyzed mouse survival during the acute phase of infection. C57BL/6 mice infected with Wh3Δ*mic1* strain showed 2–3 days longer survival compared with Wh3-infected mice that all succumbed to infection by day 9, reflecting an attenuated virulence upon deletion of *mic1* in Wh3. Additionally, mice infected with Wh6 strain died less than 60%, and no cyst was found in the brain of the undead infected mice (Fig. 2A).

The difference in the survival phenotype suggests further investigation of the acute phase of the infection. We next tested the effect of TgMIC1 on parasite burden in vivo, and 5 days postinfection, the number of tachyzoites in the blood of the Wh3Δ*mic1*-infected mice was significantly lower than that of the Wh3-infected control mice. Similar results were obtained in the intestine, brain, and spleen, as manifested by the lower transcript levels of Tg529 in mice infected with the Wh3Δ*mic1*, reflecting less burden of this parasite to these organs (Fig. 2B). However, no significant differences were observed between the parasite loads of Wh6- and Wh3Δ*mic1*-infected mice in the intestine and brain, suggesting that MIC1 may be tissue-biased or the result of differences in the expression of certain receptors in these tissues, which we did not investigate further here.

To elucidate the serum inflammation levels in mice infected with different strains, we measured the levels of IL-1β, IL-18, IL-10, and IFN-γ. The levels of cytokines in the Wh6-infected and Wh3Δ*mic1*-infected mice decreased compared with the Wh3 infection group. There was no significant difference in cytokine expression levels in the serum of mice in the Wh6-infected group compared with the Wh3Δ*mic1*-infected group. Additionally, the IL-18 level decreased most significantly in the Wh3Δ*mic1* infection group, and IL-1β also decreased to a certain extent (Fig. 2C). The reduction in inflammatory factors, as well as the parasite burden, may explain this attenuated virulence during the acute phase of the infection.

### TgMIC1 induces pyroptosis in BMDM

**Fig. 3:**
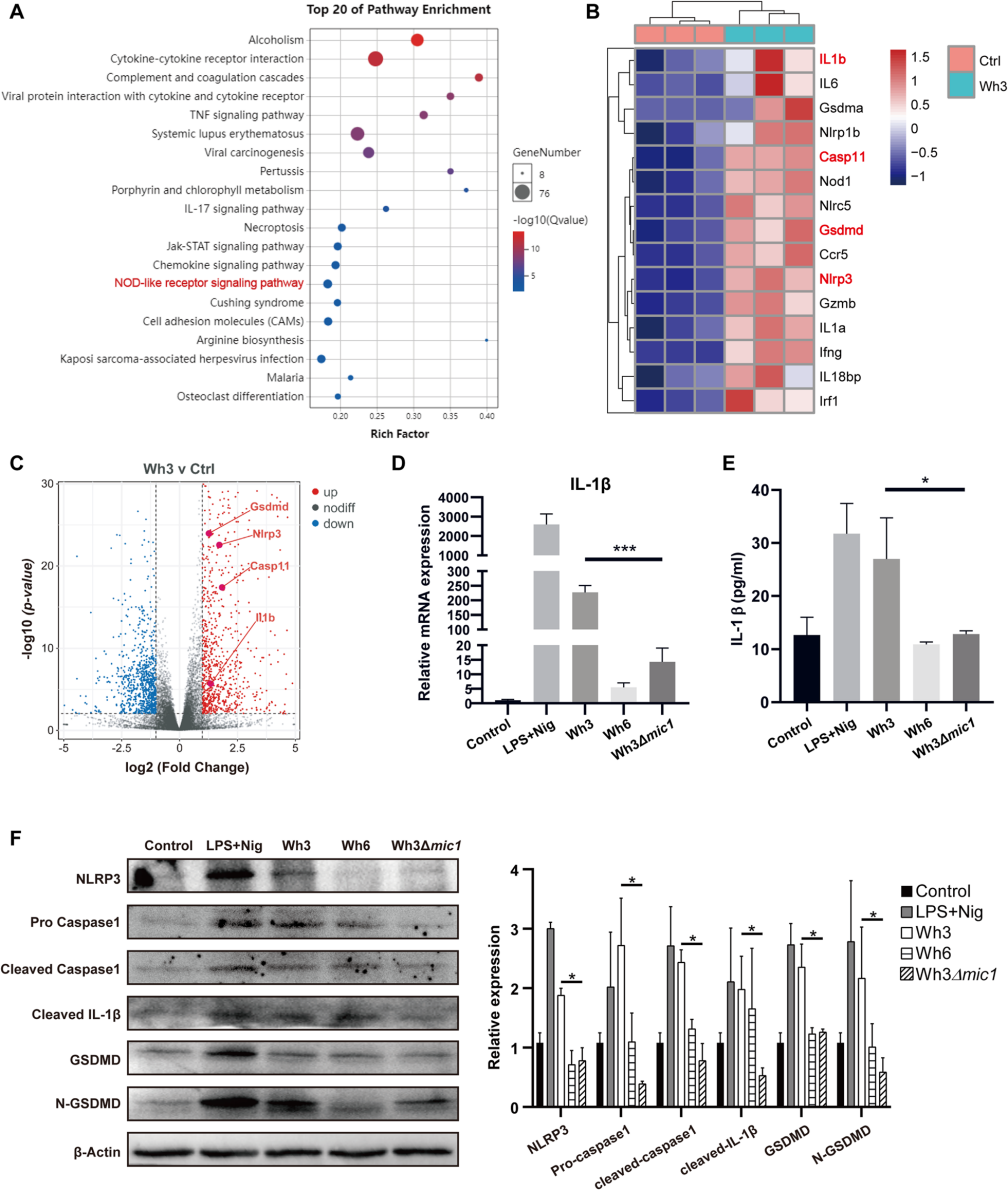
TgMIC1 can induce pyroptosis in BMDM. **A** Enrichment of gene pathways. **B** Volcano plot showing the difference in gene expression between the control group and the Wh3 infection group. Example genes are marked in red. **C** Significantly differentially expressed genes between the two groups were selected according to interest and plotted in a heat map using complete linkage clustering of the Euclidean distance matrix for all samples. **D** The mRNA expression level of IL-1β was detected in BMDMs by qRT-PCR. One-way ANOVA was used for statistical analyses. Statistical differences are expressed as *P < 0.05 *n* = 3. **E** The cell culture supernatant was collected for ELISA detection. One-way ANOVA was used for statistical analyses. Statistical differences are expressed as *P < 0.05 (*n* = 3). **F** Detecting pyroptosis-related proteins in BMDMs infected by tachyzoites using WB. Data are presented as the mean ± standard error of the mean (SEM) of each group (*n* = 3) (three independent experiments), and a *t*-test was used for statistical analysis. * *P* < 0.05

To identify the immune characteristics induced by Chinese 1 *Toxoplasma* infection in the host and gain insights into the role of TgMIC1 in immune regulation, we infected mice with the Wh3 strain and conducted RNA-seq analysis on spleen cells obtained from both infected and uninfected mice. Pathway enrichment analysis revealed a significant upregulation of the inflammatory pathway in mice infected with the Wh3 strain compared with the uninfected group. Additionally, the NOD-like receptor signaling pathway, closely associated with pyroptosis, was also significantly upregulated (Fig. 3A). Through the analysis of differentially expressed genes, it was observed that the two cell groups exhibited significant separation, signifying substantial differences before and after infection (Fig. 3C). Subsequent examination of the differentially expressed genes revealed a significant enrichment of pyroptosis-related genes, including IL-1β, caspase-11, GSDMD, and NLRP3, in the spleen cells of infected mice (Fig. 3B).

To investigate the role of TgMIC1 in immune regulation, BMDMs were infected with the Wh3, Wh6, and Wh3Δ*mic1* strains. Subsequent qRT-PCR analysis of cellular RNA revealed a significant downregulation of pro-IL-1β mRNA expression in Wh3Δ*mic1*-infected BMDMs compared with Wh3-infected BMDMs (Fig. 3D). Upon ELISA analysis of the cell culture supernatant, it was evident that IL-1β expression was significantly higher in BMDMs infected with the Wh3 strain than in those infected with the Wh3Δ*mic1* strain (Fig. 3E). Subsequent detection and analysis of protein expression changes revealed a significant downregulation of GSDMD, NLRP3, and caspase-1 in Wh3Δ*mic1*-infected BMDMs compared with Wh3 infected BMDMs. Additionally, the protein expression levels of pro-caspase-1 and cleaved caspase-1 were slightly lower in the Wh6 infected group (Fig. 3F), potentially linked to the low expression of MIC1 by Wh6.

### TgMIC1 induces pyroptosis in BMDMs through the TLR4/NLRP3 pathway

**Fig. 4:**
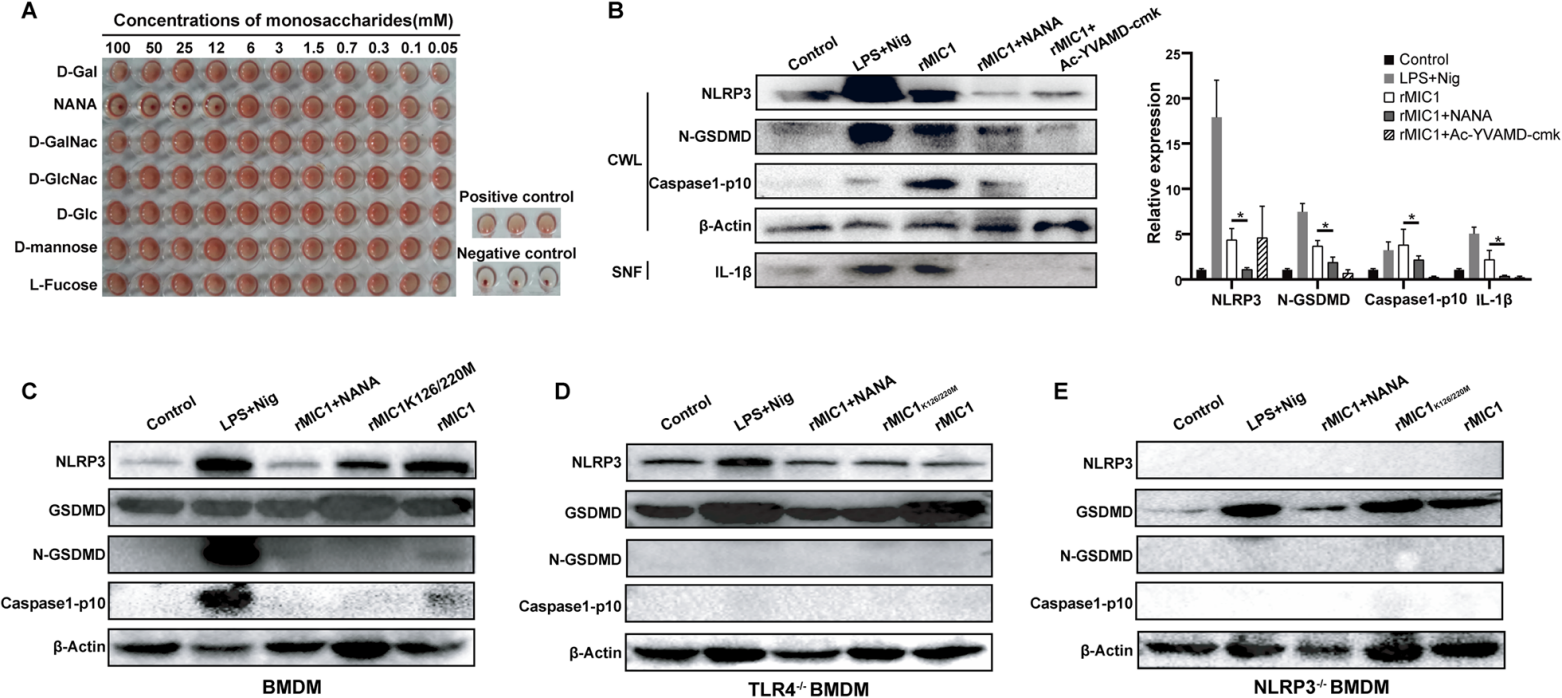
TgMIC1 induces pyroptosis in BMDMs through the TLR4/NLRP3 pathway. **A** Hemagglutination activity of rMIC1 detected by different concentrations of d-Gal, NANA, d-GalNac, d-GlcNac, d-mannose, and l-fucose. WB detection of pyroptosis-related proteins in (**B**) cell lysates and supernatants, **C** BMDMs, **D** TLR4^−/−^ BMDM, and **E** NLRP3^−/−^ BMDM. Data are presented as the mean ± standard error of the mean (SEM) of each group (*n* = 3) (three independent experiments), and a *t*-test was used for statistical analysis. * *P* < 0.05

Subsequently, rMIC1 was utilized to perform hemagglutination inhibition experiments, confirming its sialic acid lectin activity (Fig. 4A). Through WB detection, a significant upregulation was observed in the expression of NLRP3, N-GSDMD, and caspase-1 p10, along with the detection of IL-1β in the cell supernatant, when only rMIC1 was used for stimulation compared with the control group. Moreover, pretreatment of BMDMs with NANA inhibited the increase in protein expression levels induced by rMIC1, leading to the nondetection of IL-1β in the cell supernatant. Furthermore, pretreating BMDMs with Ac-YVAMD-cmk to inhibit caspase-1 prior to rMIC1 stimulation resulted in the absence of activated caspase-1, a significant downregulation of N-GSDMD, and the nondetection of IL-1β in the cell supernatant (Fig. 4B).

To further explore how MIC1 regulates pyroptosis, we double-mutated sites 126 and 220 of rMIC1 to inactivate the MAR domain of rMIC1-T126A/T220A. In addition, we extracted the BMDMs from NLRP3^−/−^ and TLR4^−/−^ mice to obtain NLRP3^−/−^ BMDMs and TLR4^−/−^ BMDMs. As shown in Fig. 4, BMDMs stimulated by rMIC1 cleaved GSDMD to form N-GSDMD and activated caspase-1. Conversely, rMIC1-T126A/T220A, lacking binding sites to *N*-glycan in TLR4, was unable to activate caspase-1 and GSDMD in BMDMs. Moreover, when rMIC1 was used to stimulate TLR4^−/−^ and NLRP3^−/−^ BMDMs, neither caspase-1 nor GSDMD could be activated, and rMIC1 failed to upregulate the expression of NLRP3 in TLR4-deficient BMDMs (Fig. 4C, D, E).

## Discussion

The Chinese 1 genotype of *T. gondii* is prevalent in China. Previous studies have confirmed differential expression of TgMIC1 between the Wh3 and Wh6 strains [28]. The Wh3 strain of Chinese 1 *T. gondii* induces a strong inflammatory response in the host, often leading to host mortality, while the Wh6 strain tends to establish a chronic infection within the host. These two strains display significant differences in virulence in mice [15]. Despite extensive research on the differences in virulence among the type I, II, and III strains of the archetypal clonal lineages [29], the factors influencing the differences in virulence between Wh3 and Wh6 remain unclear. In this study, we observed a reduction in invasion capability, parasite load, and levels of inflammatory cytokines in TgMIC1-deleted Wh3 tachyzoites compared with the wild-type Wh3. These findings indicate that TgMIC1 facilitates the proliferation of Chinese 1 *Toxoplasma* in mice, leading to increased production of proinflammatory factors and heightened virulence. Given that factors such as invasiveness, intracellular replication capacity, and the nature of the induced immune response collectively contribute to *T. gondii*’s virulence, we infer that the elevated expression of TgMIC1 in the Wh3 strain may partially account for its heightened virulence relative to Wh6. Inconsistent to our research findings, a prior study reported that the deletion of *mic1* in the RH strain did not result in a reduction of parasite burden in mice, as compared with mice infected with wild-type RH tachyzoites [30]. This discrepancy may stem from the RH strain’s higher proliferation rate than that of Wh3, rendering the impact of *mic1* deletion on parasite load insignificant. Alternatively, it is plausible that TgMIC1 collaborates with unidentified factors to assume distinct roles within the RH strain.

TgMIC1, one of the earliest identified MICs in *T. gondii*, is a soluble adhesion protein typically facilitates the invasion of the parasite by forming complexes with TgMIC4 and TgMIC6 [31]. Subsequently, following the invasion of *T. gondii* into target cells, this complex is released into the tissue with the aid of proteolytic enzymes [32, 33]. While extensive research has focused on the role of MIC1 in invasion, recent studies have also suggested its involvement in immunomodulation during *T. gondii* infection, necessitating further in-depth investigation of its mechanism of action. Previous structural analysis has shown that TgMIC1 contains two MAR domains with sialic acid lectin properties that selectively target α (2–3)-sialyl residues linked to β-galactosides [22–24, 34]. We established the lectin properties of TgMIC1 from Chinese 1 in vitro using rMIC1. Our results showed that the coagulation phenomenon triggered by rMIC1 can be specifically blocked by NANA but not by d-Gal, d-GalNac, d-GlcNac, d-mannose and l-fucose. The glycan–lectin interaction can initiate the activation of the receptor [35].

Previous research has demonstrated that TgMIC1 interacts with TLR2/TLR4, leading to the activation of the NF-κB signaling pathway and subsequent induction of interleukin-12 (IL-12) and interferon-gamma (IFN-γ) [30, 36, 37]. Additionally, TgMIC1 plays a role in regulating the expression of interleukin-10 (IL-10) in macrophages through TLR4 endocytosis [35]. In our current investigation, we noted a notable reduction in IFN-γ and IL-10 levels following the deletion of *mic1* in Wh3, aligning with prior findings. Furthermore, a more pronounced decrease in IL-1β and IL-18, cytokines linked to cell pyroptosis, was observed, suggesting the participation of TgMIC1 in modulating the host cell pyroptosis signaling pathway.

Pyroptosis, a regulated form of cell death, is governed by multiple inflammasomes, which are intracellular multiprotein complexes comprising NLRP1, NLRC4, AIM2, and others [38]. In the context of pyroptosis, NLRP3 is generally recognized as the primary inflammasome [39]. Our study validated that the deletion of *mic1* resulted in the decreased expression of NLRP3 in BMDMs and notably reduced the activation of caspase-1 and GSDMD in BMDMs. Furthermore, stimulation of BMDMs with rMIC1 alone significantly increased the expression of NLRP3, N-GSDMD, and caspase-1 p10, as confirmed by WB assay. To investigate the impact of TgMIC1 binding to TLR-like receptors on host immune responses, we exposed BMDMs to NANA and generated rMIC1-T1226A/T220A mutants with alterations in the carbohydrate-recognition domains (CRD). Our findings suggest that the interaction between sialic acid and TgMIC1 played a pivotal role in modulating pyroptosis. Pyroptosis can be classified into classical and nonclassical types, depending on whether it is triggered by caspase-1 activation [40]. In our study, we utilized the caspase-1 inhibitor Ac-YVAMD-cmk in BMDMs and observed that rMIC1 failed to induce pyroptosis following caspase-1 inhibition. Furthermore, we conducted experiments using BMDMs from TLR4 knockout mice and NLRP3 knockout mice and found that TgMIC1 primarily modulates macrophage pyroptosis through TLR4 and NLRP3. Although it has been demonstrated that pyroptosis and inflammation can restrict the growth of *toxoplasma*, intense inflammatory responses can also lead to acute death in mice [41]. The absence of *mic1* results in impaired invasion ability of *Toxoplasma*, but its capacity to induce pyroptosis and immune responses is also weakened. This may explain why, despite an extended survival period, mice still succumb to death.

## Conclusions

TgMIC1 is involved not only in the invasion of the parasite but also in the regulation of macrophage pyroptosis through the TLR4/NLRP3 pathway, and its differential expression in the Wh3 and Wh6 strains affects virulence to a certain extent. This investigation indicates that TgMIC1 serves diverse functions in *T. gondii* infection, thereby enhancing comprehension of the immune regulatory mechanisms of the parasite.

## Supplementary Information

Additional file 1

## Data Availability

The datasets generated and analyzed during the current study are available in the NCBI repository, https://www.ncbi.nlm.nih.gov/bioproject/PRJNA1117525.

## Abbreviations

BMDMs: Bone marrow-derived macrophages
DMEM: Dulbecco’s modified Eagle medium
gRNA: Guide RNA
HFF: Human foreskin fibroblast
MIC1: Microneme protein1
MPP1: Microneme processing protease 1
NLRP3: NOD-like receptor pyrin domain-containing protein 3
rMIC1: Recombinant TgMIC1 proteins
ROM: Rhomboid
*T. gondii*: *Toxoplasma gondii*
Wh3: TgCtwh3
Wh6: TgCtwh6
